# Comparison of Transversus Abdominis Plane Infiltration with Liposomal Bupivacaine versus Continuous Epidural Analgesia versus Intravenous Opioid Analgesia

**DOI:** 10.1371/journal.pone.0153675

**Published:** 2016-04-15

**Authors:** Sabry Ayad, Rovnat Babazade, Hesham Elsharkawy, Vinayak Nadar, Chetan Lokhande, Natalya Makarova, Rashi Khanna, Daniel I. Sessler, Alparslan Turan

**Affiliations:** 1 Department of Anesthesiology and Pain Management, Fairview Hospital; Department of Outcomes Research, Cleveland Clinic, Anesthesiology Institute Cleveland, Ohio; 2 Department of Anesthesiology, University of Texas Medical Branch, Galveston, Texas and Department of Outcomes Research, Cleveland Clinic, Cleveland, Ohio; 3 Departments of General Anesthesiology and Outcomes Research, Cleveland Clinic, Anesthesiology Institute, Cleveland, Ohio; 4 Department of Outcomes Research, Cleveland Clinic, Anesthesiology Institute, Cleveland, Ohio; 5 Departments of Qualitative Health Sciences and Outcomes Research, Cleveland Clinic, Anesthesiology Institute, Cleveland, Ohio; University of Colorado, UNITED STATES

## Abstract

Epidural analgesia is considered the standard of care but cannot be provided to all patients Liposomal bupivacaine has been approved for field blocks such as transversus abdominis plane (TAP) blocks but has not been clinically compared against other modalities. In this retrospective propensity matched cohort study we thus tested the primary hypothesis that TAP infiltration are noninferior (not worse) to continuous epidural analgesia and superior (better) to intravenous opioid analgesia in patients recovering from major lower abdominal surgery. 318 patients were propensity matched on 18 potential factors among three groups (106 per group): 1) TAP infiltration with bupivacaine liposome; 2) continuous Epidural analgesia with plain bupivacaine; and; 3) intravenous patient-controlled analgesia (IV PCA). We claimed TAP noninferior (not worse) over Epidural if TAP was noninferior (not worse) on total morphine-equivalent opioid and time-weighted average pain score (10-point scale) within first 72 hours after surgery with noninferiority deltas of 1 (10-point scale) for pain and an increase less of 20% in the mean morphine equivalent opioid consumption. We claimed TAP or Epidural groups superior (better) over IV PCA if TAP or Epidural was superior on opioid consumption and at least noninferior on pain outcome. Multivariable linear regressions within the propensity-matched cohorts were used to model total morphine-equivalent opioid dose and time-weighted average pain score within first 72 hours after surgery; joint hypothesis framework was used for formal testing. TAP infiltration were noninferior to Epidural on both primary outcomes (p<0.001). TAP infiltration were noninferior to IV PCA on pain scores (p = 0.001) but we did not find superiority on opioid consumption (p = 0.37). We did not find noninferiority of Epidural over IV PCA on pain scores (P = 0.13) and nor did we find superiority on opioid consumption (P = 0.98). TAP infiltration with liposomal bupivacaine and continuous epidural analgesia were similar in terms of pain and opioid consumption, and not worse in pain compared with IV PCA. TAP infiltrations might be a reasonable alternative to epidural analgesia in abdominal surgical patients. A large randomized trial comparing these techniques is justified.

## Introduction

Pain management after major abdominal surgery remains challenging. The best-accepted analgesic approach is continuous epidural analgesia which is generally thought to be considerably more effective than intravenous patient-controlled opioid analgesia (IV PCA) [1, 2]. However, epidural analgesia can cause hemodynamic instability, along with motor weakness and consequent restriction of ambulation. Furthermore, epidural catheter placement can be time consuming and challenging, especially in obese patients and those with spinal pathology. And finally, epidural catheter insertion is contraindicated in anti-coagulated patients [3].

The Transversus Abdominis Plane (TAP) infiltration is an alternative approach to providing postoperative analgesia to the anterior abdominal wall [4]. TAP infiltration is relatively easy to perform, generally safe, and can be performed in patients who are anti-coagulated [5, 6]. TAP infiltration can be performed as a single injection, or a catheter can be inserted for continuous local anesthetic infusion [7, 8]. Single-shot TAP infiltration with conventional local anesthetics do not last sufficiently long to provide effective postoperative analgesia. However, recently developed liposomal bupivacaine provides much longer-lasting analgesia than plain bupivacaine [9].

It would be attractive if single-shot TAP infiltration with liposomal bupivacaine were as effective as continuous epidural analgesia. We, therefore, tested the primary hypothesis that TAP infiltration with single-shot liposomal bupivacaine is noninferior (not worse) to continuous epidural analgesia in patients recovering from major lower abdominal surgery. Secondarily, we tested the hypotheses that both TAP infiltration and epidural analgesia provide better postoperative analgesia than IV PCA. We also evaluated the association between analgesic strategy and hospital length-of-stay, postoperative paralytic ileus, steroid administration, non-steroidal anti-inflammatory (NSAID) and antiemetic medication use, and time to first rescue opioid postoperatively.

## Methods

With approval of the Cleveland Clinic Research Advisory Committee of Anesthesiology Institute (07/16/2014) and the Cleveland Clinic Institutional Review Board (14–857, 7/29/2014), we obtained data on major [10] lower-abdominal surgeries, all with general anesthesia, in adults (>18 years old) at the Cleveland Clinic Fairview and Main Campus hospitals between January 2012 and July 2014. Requirement for written informed consent was waived by the Cleveland Clinic Institutional Review Board. Patient information was anonymized and de-identified prior to analysis. The patients with ASA physical status V and above and patients that received TAP block with medication other than liposomal bupivacaine were excluded. Surgeries with missing baseline and potential confounding measurements (body mass index and ASA physical status) were excluded. The most recent surgical procedure was used for patients in whom multiple major lower abdominal surgeries were found in our registry.

The Cleveland Clinic Perioperative Health Documentation System contains information about all patients who have non-cardiac surgery since May of 2005 at Cleveland Clinic’s main campus and since August 2013 at Cleveland Clinic Fairview campus. It integrates preoperative variables (demographics, conditions, etc.), intraoperative variables (via the Anesthesia Record Keeping system) and postoperative outcomes (by linking to the Cleveland Clinic billing data systems).

Demographic and baseline data obtained from the registry were augmented by manually-encoded additional postoperative medication data obtained from eligible patients’ electronic medical records.

We considered three primary postoperative analgesic strategies after lower abdominal surgery under general anesthesia. (1) TAP infiltration: a bilateral TAP infiltration was performed preoperatively, and a total of 40 mL solution was injected, consisting of 20 mL (5 mg/mL) bupivacaine and 20 mL (13.3 mg/mL) liposomal bupivacaine (Exparel, Pacira Pharmaceuticals, Parsippany, NJ, USA). (2) Epidural analgesia: an epidural catheter was inserted preoperatively and mainly started intraoperatively. The epidural solution contained fentanyl 2 mcg/mL and bupivacaine 0.1%, and was given at a basal rate of 5–7 mL/hour, with demand boluses of 6 mL and lockout interval time of 15 minutes. (3) IV PCA: patients were given IV PCA with hydromorphone 0.5 mg/mL, fentanyl 20 mcg/mL, or morphine 1 mg/mL. The rescue analgesia was given as per hospital policy in all the three groups (ordered by clinicians if pain score (NRS) ≥ 4) and included opioids, NSAID medication. Following the emergence administration of the first analgesic medication was counted as a first rescue analgesic. If rescue analgesia included opioid medication it was reflected in the outcome ‘Total opioid IV morphine equivalent dose until 72 hours’. All rescue medications including times, dates, pre and post administration pain score documentation are in electronic medical record (EPIC). TAP and Epidural groups were also given supplemental IV PCA with similar settings for postoperative pain management.

Our two primary outcomes were total postoperative intravenous (IV) morphine-equivalent dose of opioid and time-weighted average numeric rating scale (NRS) pain scores. Both were evaluated over 72 postoperative hours or until hospital discharge, whichever came first. All procedures were performed by anesthesiologist. Postoperative pain management, follow up completed by acute pain service and surgical team. Post-anesthesia care unit and floor nurses were assessed and recorded 10 point numeric rating pain scores. NRS pain scores were obtained by nurses, per routine, roughly every four hours. Postoperative opioid medications were obtained from electronic medical records and were converted into IV morphine-equivalent doses using the opioid conversion rates from Table 1 [11, 12].

**Table 1:** Opioid conversion doses.

Medication name	Route	Units	Equivalent Dose
Morphine	IV	mg	10
Morphine	Oral	mg	30
Fentanyl	IV	mg	0.1
Fentanyl	IV	mcg	0.1
Fentanyl	patch	mg	0.1
Fentanyl	patch	mcg	100
Fentanyl	epidural PCA	mg	0.1
Fentanyl	oral	mg	0.229
Fentanyl	oral	mcg	229
Alfentanil	IV	mg	0.67
Meperidine	IV	mg	75
Meperidine	oral	mg	333
Demerol	IV	mg	75
Oxycodone	oral	mg	20
Percocet	oral	mg	20
Percocet 5/325	oral	tabs	6
Darvocet	oral	tabs	1
Propoxyphene	oral	tabs	1
Oxycontin	oral	mg	20
Hydrocodone	oral	mg	30
Vicodin 5/500	oral	tabs	6
Vicodin 7.5/500	oral	tabs	4
Tramadol	oral	mg	150
Hydromorphone	IV	mg	1.5
Hydromorphone	oral	mg	7
Dilaudid	IV	mg	1.5
Dilaudid	oral	mg	7
Remifentanil	IV	mg	0.1
Sufentanil	IV	mg	0.01
Methadone	oral	mg	20
Codeine	oral	mg	200

PCA = Patient-controlled analgesia; IV = intravenous.

Sex, race, American Society of Anesthesiologists (ASA) Physical Status, history of diabetes, chronic pain syndrome, history of chronic opioid use, preoperative steroid and statins use, year of surgery, elective or emergency surgical status, type of the surgery, surgical approach (open vs. laparoscopic procedure), intraoperative administration of opioids, nonsteroidal anti-inflammatory drugs (NSAID), and steroid were considered for confounding adjustment and coded as categorical or binary variables. Age, body mass index, and duration of surgery were also considered for confounding adjustment, and coded as continuous variables.

### Statistical analysis

Patients receiving TAP infiltration were matched to patients receiving Epidural and patients receiving only IV PCA patients in a 1:1:1 ratio based on propensity scores [13]. Propensity scores (i.e., the estimated the probability of receiving TAP infiltration) were estimated for each patient using two logistic regressions; all pre-specified potential confounding variables listed above except for year of the surgery, type of the surgery and surgical approach were used in these models.

Triplets of propensity-matched patients were obtained by a three-step procedure. First, each patient who received TAP infiltration was matched to an Epidural patient. Then, each patient who received TAP infiltration was again matched, this time to a patient who received IV PCA. Finally, a filter was applied to include only those TAP infiltration patients who were successfully match to both an Epidural patient and IV PCA only patient thus resulting in the matched triplets. Matching was limited to pairs within 0.2 standard deviations of the propensity score logits (i.e., within 0.2 × 1.06 = 0.2) of one another [14].

Successfully matched triplets were restricted to those for which all three patients had common type of lower abdominal surgery (general, colorectal, or gynecologic), all three surgeries were either open or laparoscopic and occurred with one year of each other. All subsequent analyses were restricted this subset of matched patients. Potential confounding variables remaining imbalanced after matching (if difference between variables significant at the 0.05 significance level) were used for adjustment in all subsequent analyses.

SAS statistical software version 9.3 (SAS Institute, Cary, NC, USA) for 64-bit Microsoft Windows and R statistical software version 2.15.2 for 64-bit Unix operating system (The R Foundation for Statistical Computing, Vienna, Austria) were used for all statistical analyses.

#### Primary outcome

**Fig 1:**
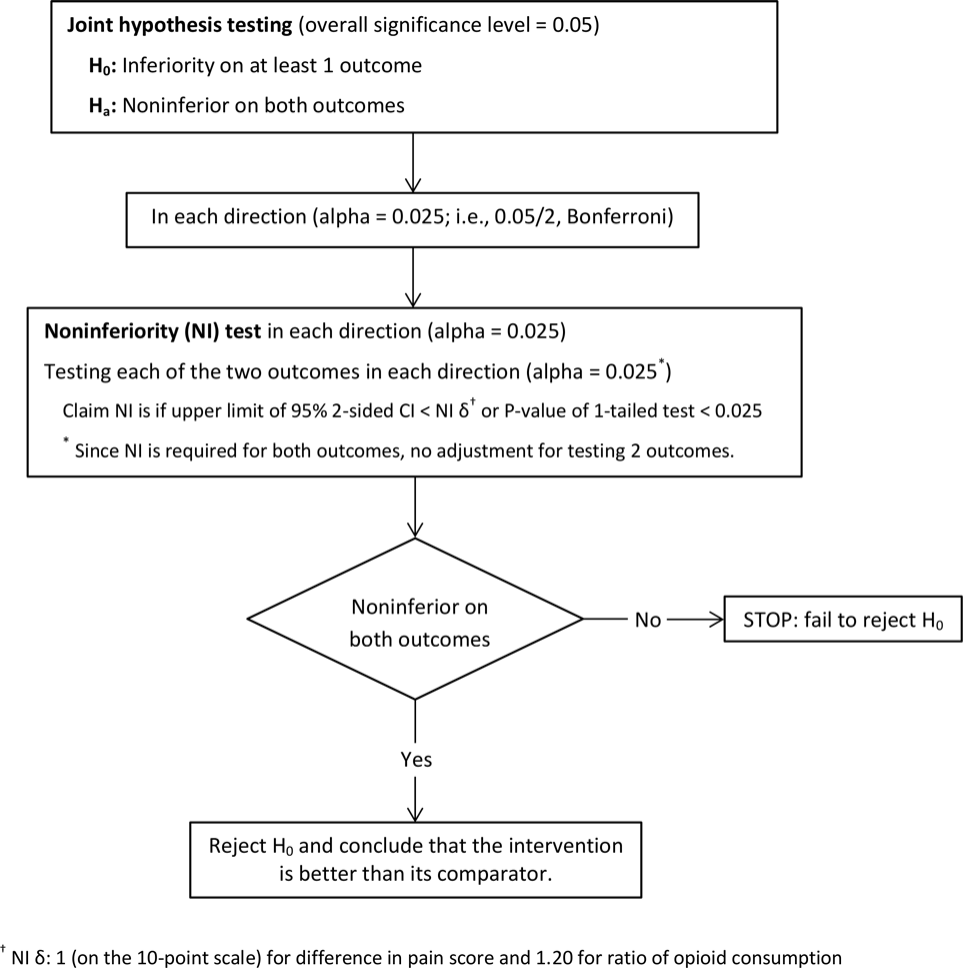
Join hypothesis testing algorithm for noninferiority on two primary outcomes.

We assessed the relative effect of the three treatment groups on postoperative pain and opioid consumption using a joint hypothesis testing framework as described in Mascha and Turan [15]. Based on the stated hypotheses, we would claim that TAP infiltration was preferred to Epidural if TAP infiltration was shown to be noninferior on both pain and opioid consumption. Conversely, in other direction, Epidural would be preferred over TAP infiltration if Epidural was shown to be noninferior on both pain and opioid consumption. We assessed noninferiority of TAP infiltration to Epidural on both time-weighted average pain score and opioid consumption within 72 hours of the surgery or hospital discharge with 1-tailed noninferiority t-tests and using pre-specified noninferiority deltas of 1 point higher on the 0–10 NRS pain scale for pain sore and an increase of 20% in the mean of opioid consumption compared to the respective reference group. The Fig 1 displays the algorithm of noninferiority testing using a joint hypothesis framework to compare TAP infiltration to Epidural each direction (TAP infiltration versus Epidural, Epidural versus TAP infiltration) on both outcomes.

Multivariable linear regression was used to model opioid consumption and pain score within the propensity-matched cohort of patients; opioid consumption was log-transformed during the modeling process. Therefore, we assessed noninferiority on opioid consumption based on the exponentiated difference in means on the log scale (i.e., the ratio of geometric means). Thus for opioid consumption, we tested the alternative hypothesis that the ratio of geometric means that there was less than 20% increase in opioid consumption with TAP infiltration than Epidural analgesia, and vice versa. For pain, we tested the alternative hypothesis that mean pain score for TAP infiltration was less than 1 point greater than with Epidural, and vice versa.

Lastly, we compared each of TAP infiltration and Epidural to IV PCA patients on total postoperative opioid consumption and pain score within first 72 hours of the surgery or until discharge. TAP infiltration or Epidural would be considered preferable to IV PCA if found superior on opioid consumption and at least noninferior on pain score (with noninferiority delta of 1). All tests were one-tailed.

Our primary hypothesis was assessed in a joint hypothesis testing framework which controlled the type I error at 0.025 (since all tests are one-sided) across all noninferiority and superiority testing [15] comparing TAP infiltration and Epidural to IV PCA groups. Throughout the testing there were no adjustment of the significance criteria for assessing both pain and opioid consumption (i.e., multiple outcomes) since significance on both outcomes was required to claim an intervention better than the comparator (i.e., an intersection-union test). However, Bonferroni correction was used for four pairwise comparisons of interest (i.e., TAP infiltration versus Epidural, Epidural versus TAP infiltration, and each of TAP infiltration and Epidural versus IV PCA), and so 0.025/4 = 0.00625 for each one-sided test. (α = 0.00625, 98.75% confidence intervals).

The average pain scores for three matched groups over first 72 hours after surgery were summarized graphically with loess regression curve (locally weighted mean curve) [16].

To address reviewer’s concerns the sensitivity analysis was performed analogous to the primary, except excluding OB/GYN, laparoscopic and emergency surgeries as well as patients with history of opioid use from the matched cohort.

#### Secondary outcomes

Patients who did not receive any rescue opioid within 72 hours of the surgery or had incomplete time to first rescue opioid administration were censored at 72 hours after surgery or at hospital discharge. The association between pain management strategies and time to first rescue opioid administration was assessed with a Cox proportional hazards model that accounts for censoring. Four separate logistic regression models were developed to model associations between study groups and each of postoperative paralytic ileus and NSAID, steroid use, and antiemetic administration.

We also assessed the association between pain management strategies and duration of postoperative hospitalization. A linear model was used with log-transformed duration of postoperative hospitalization (to meet the normality assumptions) as an outcome and pain management strategies as a predictor.

We compared the three study groups on each secondary outcome using an appropriate 2-tailed model-based Wald test. The significance level for the set of secondary outcomes were preserved at 0.05 overall by using a significance criterion of P < 0.05/6/3 = 0.0028 for each test (applying Bonferroni correction for three pairwise comparisons on each of six secondary outcomes).

#### Power consideration

Power analysis was based on the analysis of noninferiority on opioid consumption because this analysis required more patients than analysis on pain scores.

We included all available TAP infiltration patients in our analysis. After matching, there were 106 patients per group for a total of 318 patients. This sample size provided 98% power at the 0.0625 significance level to detect noninferiority of TAP infiltration to Epidural on opioid consumption assuming a noninferiority delta of ratio of means of 1.2 for opioids (less 20% increase in opioids), true ratio of means of 0.6 and a coefficient of variation (CV = SD/mean before log-transformation) of 1.5.

## Results

**Fig 2:**
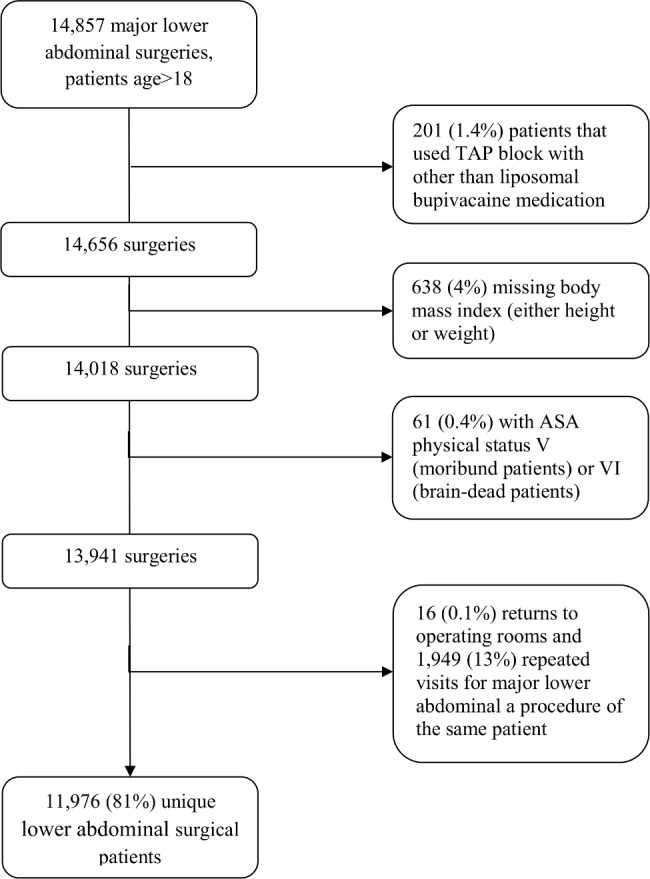
Flow chart of patient selection.

Our query of the Perioperative Health Documentation System revealed 14,857 major lower-abdominal surgeries on adults and after eliminating patients with missing baseline measurements, and repeated procedures, 11,976 patients remained, including 122 (1%) who received TAP infiltration and, 800 (7%) who received epidural, and 11,054 (92%) who received IV PCA (Fig 2).

Based on demographic and baseline characteristics, we successfully 1:1:1 matched a total of 318 patients with 106 patients per group. We thus included 87% of 122 TAP infiltration patients. Table 2 shows the group characteristics after propensity score matching; all patients’ baseline characteristics and most of surgical factors no longer significantly differed among the three groups, with exception of surgery duration. Thus, duration of surgery was included for adjustment in the primary and secondary analyses.

**Table 2:** Patients’ baseline characteristics and surgery description for three groups after 1:1:1 propensity score matching. The three groups are patients who received TAP infiltration, Epidural, or IV PCA.

Factor		TAP	Epidural	IV PCA	P-value
		(N = 106)	(N = 106)	(N = 106)	
Patients' baseline characteristics					
Age (years)		53 ± 17	52 ± 16	54 ± 17	0.60
Male Gender (vs. Female)		45 (42)	50 (47)	54 (51)	0.46
Body Mass Index (kg/m2)		26 ± 6	26 ± 6	25 ± 5	0.86
Race (%)	White	97 (92)	101 (95)	100 (94)	0.59
	Black	7 (7)	3 (3)	3 (3)	
	Others	2 (2)	2 (2)	3 (3)	
ASA Physical Status (%)	I	1 (1)	0 (0)	0 (0)	0.53
	II	35 (33)	32 (30)	32 (30)	
	III	67 (63)	73 (69)	69 (65)	
	IV	3 (3)	1 (1)	5 (5)	
Diabetes (%)		4 (4)	5 (5)	3 (3)	0.77
Chronic pain syndromes (%)		6 (6)	7 (7)	3 (3)	0.43
Steroids use (%)		0 (0)	0 (0)	1 (1)	0.37
Statins use (%)		1 (1)	0 (0)	2 (2)	0.36
History of (chronic) opioid use (%)		4 (4)	4 (4)	6 (6)	0.74
Surgery description					
Year of the surgery (%)	2012	0 (0)	3 (3)	0 (0)	0.95
	2013	62 (58)	58 (55)	62 (58)	
	2014	44 (42)	45 (42)	44 (42)	
Emergency surgery (%)		2 (2)	3 (3)	4 (4)	0.71
Type of lower abdominal surgery (%)	General	21 (20)	21 (20)	21 (20)	>0.99
	Colorectal	81 (76)	81 (76)	81 (76)	
	OB/GYN	4 (4)	4 (4)	4 (4)	
Open procedure (vs. Laparoscopic) (%)		102 (96)	102 (96)	102 (96)	>0.99
Intraoperative opioid use (%)		105 (99)	102 (96)	106 (100)	0.07
Intraoperative NSAID use (%)		7 (7)	1 (1)	5 (5)	0.11
Intraoperative Steroid use (%)		51 (48)	46 (43)	54 (51)	0.54
Duration of surgery (minutes)		147 ± 74	257 ± 131	181 ± 145	<0.001

Summary statistics were presented as ‘mean ± standard deviation’ or number of patients (%) as appropriate. Variables significant at the 0.5 level were adjusted for within multivariable regression models.

TAP = transversus abdominis plane; ASA = American Society for Anesthesiologists; OB/GYN = obstetrics and gynecology

### Primary outcomes

**Fig 3:**
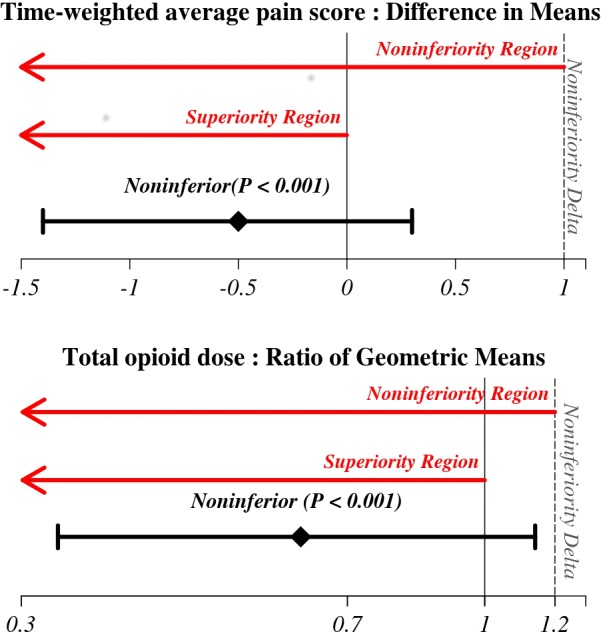
Results for comparison TAP infiltration and Epidural patients on postoperative time-weighted average pain score in the 0–10 NRS pain scale and intravenous morphine equivalent dose of opioid within 72 hours of the surgery.

**Fig 4:**
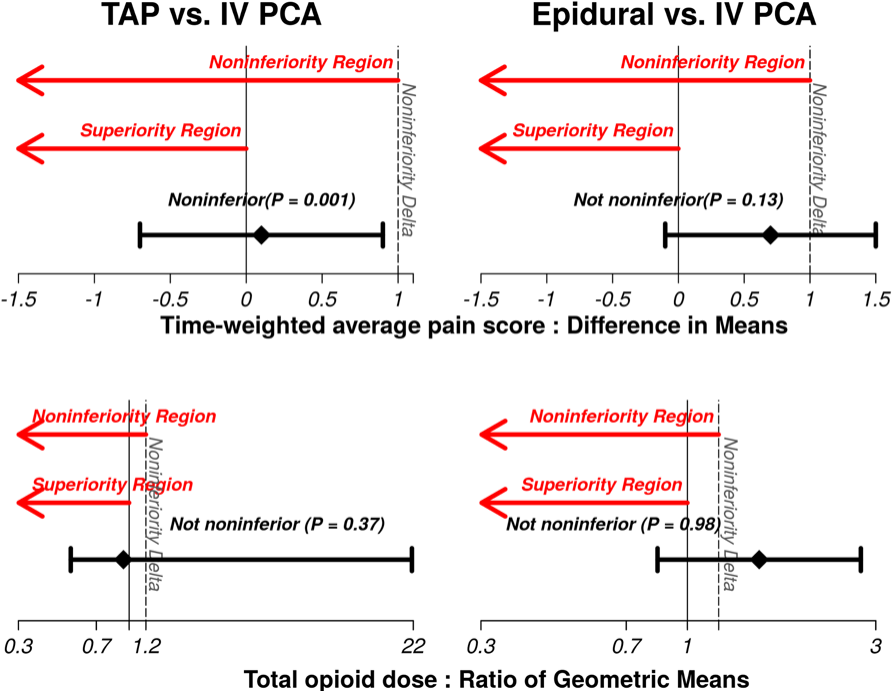
Results for two comparisons including TAP infiltration versus intravenous patient-controlled analgesia (IV PCA) and Epidural versus IV PCA on postoperative time-weighted average pain score in the 0–10 NRS pain scale and intravenous morphine equivalent dose of opioid within 72 hours of the surgery.

The TAP infiltration group was noninferior (not worse) to Epidural on both outcomes (both P<0.001 and statistically significant after application of the Bonferroni testing procedure for four pairwise comparison; significance criteria of 0.00625, Table 3 and Fig 3). The tests of Epidural versus TAP infiltration did not show noninferiority on both pain scores (P = 0.05) and opioid consumption (P = 0.93). TAP infiltration was noninferior to IV PCA group on pain scores (P = 0.001), but we did not find superiority on opioid consumption (P = 0.37, Table 3 and Fig 4). We did not find noninferiorty of Epidural over IV PCA group on pain scores (P = 0.13); nor did we find superiority on opioid consumption (P = 0.98, Table 3 and Fig 4).

**Table 3:** Results for the primary outcomes on matched patients. Observed outcomes are reported as, median [1st quartile, 3rd quartile] or ‘mean ± standard deviation’.

Outcome	TAP	Epidural	IV PCA	Estimate	P-value*
	(N = 106)	(N = 106)	(N = 106)		
				Ratio of geometric means (97.5% CI)†§	
Total opioid IV morphine equivalent dose until 72 hours of the surgery or till hospital discharge, mg	88 [28, 181]	137 [82, 246]	78 [36, 184]		
TAP vs. Epidural				0.62 (0.33, 1.14)	< .001^#^*
Epidural vs. TAP				1.62 (0.88, 3.00)	0.93^#^
TAP vs. IV PCA				0.94 (0.53, 21.67)	0.37^&^
Epidural vs. IV PCA				1.52 (0.84, 2.75)	0.98^&^
				Difference in means (97.5% CI)† ‡	
Time-weighted average NRS pain score until 72 hours of the surgery or hospital discharge	4.0 ± 1.7	4.3 ± 1.8	3.8 ± 2.0		
TAP vs. Epidural				-0.5 (-1.4, 0.3)	< .001^#^*
TAP vs. Epidural				0.5 (-0.3, 1.4)	0.05^#^
TAP vs. IV PCA				0.1(-0.7, 0.9)	0.001^#^*
Epidural vs. IV PCA				0.7 (-0.1, 1.5)	0.13^#^

TAP = Transversus Abdominis Plane; NRS = Numeric Rating Scale; CI = confidence interval; IV PCA = Intravenous Patient-Controlled Analgesia.

†Confidence limits reflect the Bonferroni adjustment for multiple comparisons in order to maintain an overall 2.5% Type I error rate for the primary outcomes.

^#^ P-value corresponds to 1-tailed noninferiority t-tests and uses pre-specified noninferiority deltas of 1 point higher on the 0–10 NRS pain scale for pain sore and an increase of 20% in the geometric mean opioid consumption (ratio of geometric means < 1.2) compared to the respective reference group.

& P-value corresponds to 1-tailed superiority t-tests.

* Significant P–value is less than 0.00625 (i.e., 0.025/4 = 0.00625) for the primary outcomes.

§ Linear model used on log-transform data and the ratio of geometric means were reported.

^‡^ Linear model used and difference in means are reported

**Fig 5:**
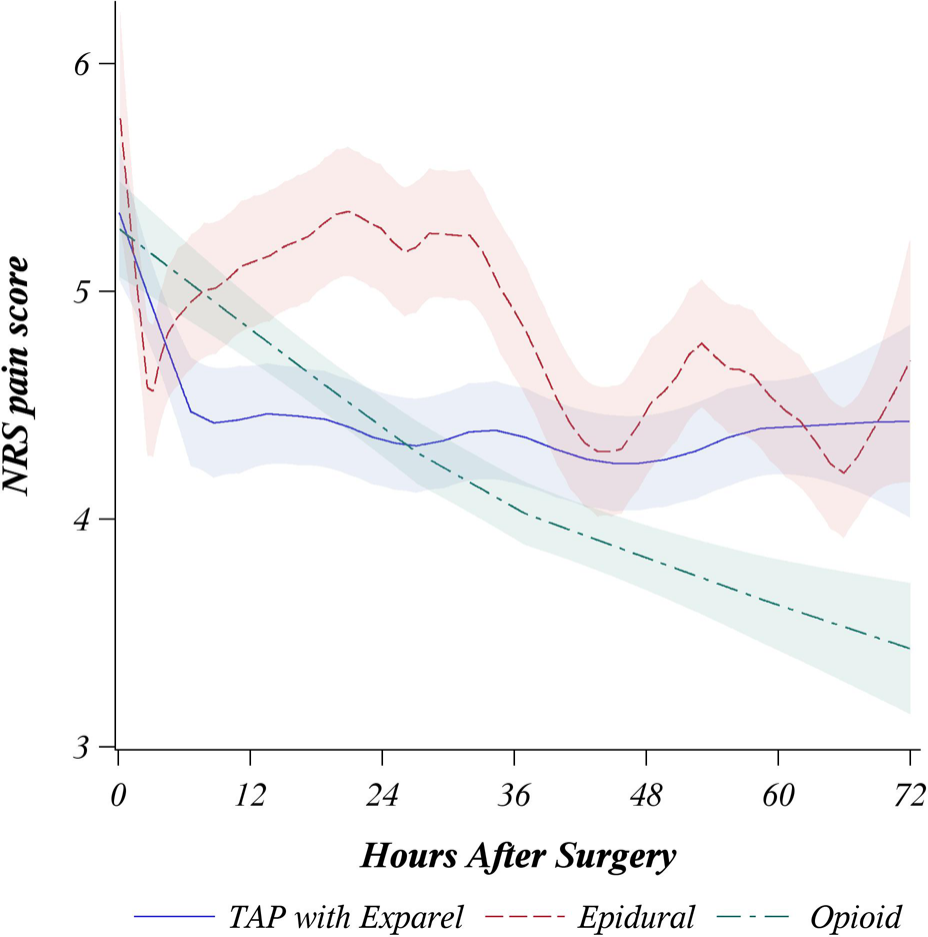
Loess curves (locally weighted mean curve) by treatment group describing mean pain scores first 72 hours after the surgery with approximate 95% confidence interval.

Summarizing our primary outcomes, TAP infiltration and Epidural were noninferior on both pain and opioid use. Neither TAP infiltration nor Epidural were preferable to IV PCA since superiority was not found on the opioid outcome and Epidural was not noninferior to IV PCA group on pain scores. The pain score loess curves over first 72 hours after surgery with approximate 95% confidence interval are shown by study groups in Fig 5. Postoperative opioid doses are presented in Table 4.

**Table 4:** Opioid administration within 72 hours of the surgery or till hospital discharge by medication.

Opioid Type	Route	TAP	Epidural	IV PCA
Fentanyl (mcg)	IV/Epi	38	97	59
Fentanyl (mcg)	Patch	200	600	275
Hydrocodone (mg)	Oral	275	265	85
Hydromorphone (mg)	IV	1,487	1,575	1,143
Hydromorphone(mg)	Oral	28	22	
Meperidine (mg)	IV	75	50	75
Morphine (mg)	IV	126	438	141
Morphine (mg)	Oral	75	120	135
Oxycodone (mg)	Oral	1,250	2,639	1,495

IV: intravenous; Epi: Epidural. TAP = Transversus Abdominis Plane

The post-hoc sensitivity analysis based on subset of the matched patients excluding OB/GYN, laparoscopic and emergency surgeries as well as patients on opioids were consistent with the results on the primary models showing a TAP block with Liposomal bupivacaine was not worse in pain and opioid consumption within 72 hours of the surgery comparing to epidural technique.

### Secondary outcomes

Secondary outcomes are summarized in Table 5. We found a significant difference between groups on one secondary outcome, duration of postoperative hospitalization. Epidural patients stayed in the hospital after the surgery 40% (95% confidence interval (0, 100%)) longer in comparison to opioid (P<0.001) patients. The incidence of postoperative paralytic ileus, steroid use, NSAID and antiemetic administration, and time to first rescue opioid administration did not differ significantly among the three study groups.

**Table 5:** Results for the secondary outcomes on matched patients. Observed outcomes are reported as, median [1st quartile, 3^rd^ quartile] or number of patients (%) as appropriate.

Outcome	TAP	Epidural	IV PCA	Estimate	P-value*
	(N = 106)	(N = 106)	(N = 106)		
				Hazard Ratio (95% CI)†§	
Time to first rescue opioid administration, minutes	44 [34, 61]	51 [37, 68]	42 [36, 60]		
TAP vs. Epidural				0.8(0.5, 1.3)	0.23
TAP vs. IV PCA				0.9 (0.6, 1.3)	0.37
Epidural vs. IV PCA				1.1 (0.7, 1.6)	0.71
				Odds Ratio (95% CI)† ‡	
Postoperative NSAID use	33 (31%)	30 (28%)	28 (26%)		
TAP vs. Epidural				1.0 (0.4, 2.6)	0.93
TAP vs. IV PCA				1.2 (0.5, 3.0)	0.54
Epidural vs. Opioids				1.2 (0.5, 3.2)	0.51
Postoperative steroid use	10 (9%)	11 (10%)	13 (12%)		
TAP vs. Epidural				1.2 (0.3, 5.2)	0.72
TAP vs. IV PCA				0.8 (0.2, 3.2)	0.69
Epidural vs. IV PCA				0.7 (0.2, 2.7)	0.43
Postoperative antiemetic medications use	58 (55%)	58 (55%)	60 (57%)		
TAP vs. Epidural				1.0 (0.4, 2.5)	0.88
TAP vs. IV PCA				0.9 (0.4, 2.2)	0.82
Epidural vs. IV PCA				0.9 (0.4, 2.1)	0.71
Postoperative paralytic ileus	14 (13%)	16 (15%)	9 (8%)		
TAP vs. Epidural				0.8 (0.2, 2.7)	0.52
TAP vs. IV PCA				1.6 (0.4, 6.2)	0.31
Epidural vs. IV PCA				2.1 (0.5, 8.3)	0.11
				Ratio of Geometric Means (95% CI)† &	
Duration of postoperative hospitalization, days	3.9 [2.8, 6.9]	6.1 [5.0, 9.3]	4.1 [3.0, 7.3]		
TAP vs. Epidural				0.7 (0.5, 1.1)	0.005
TAP vs. IV PCA				1.1 (0.8, 1.5)	0.58
Epidural vs. IV PCA				1.4 (1.0, 2.0)	< .001^#^

TAP = Transversus Abdominis Plane; CI = confidence interval; NSAID = Nonsteroidal anti-inflammatory drugs.

†Confidence limits reflect the Bonferroni adjustment for multiple comparisons in order to maintain an

* P-values correspond to 2-tailed Wald superiority tests.

Epidural local anesthetic administration was interrupted because of hemodynamic instability 10 times in 9 Epidural patients (8% of total 106 Epidural patients).

## Discussion

TAP infiltration with liposomal bupivacaine was non-inferior to epidural catheters for both pain and opioid consumption. This is a curious result since TAP infiltration provide coverage for somatic pain only [4], while epidural analgesia bocks both visceral and somatic pain [17]. The relative contributions of each pain type remains unclear, but our results suggest that the somatic pain contributes most after abdominal surgery.

There have been only a few comparisons between TAP infiltration and epidural analgesia. Kadam et al, in a retrospective review, compared TAP and thoracic epidural catheter [18] and Niraj et al. compared the analgesic effects of bilateral subcostal TAP catheters and epidural catheters in patients having upper-abdominal surgery [19]. Recently, Ganapathy et al, compared the analgesic effects of continuous bilateral lateral-to-medial approach TAP catheters and thoracic epidural analgesia in patients undergoing laparotomy [20]. All concluded that TAP and epidural infiltration provide comparable analgesia. But in contrast to our findings, they also report that more supplemental opioid was used in TAP patients. Each of these studies was marginally powered, though, which limits firm conclusions about the relative efficacy of TAP and epidural infiltration after abdominal surgery. There are currently no adequately powered randomized trials comparing TAP infiltration and epidural analgesia—and such studies are clearly needed.

In distinct contrast to our expectations, neither TAP infiltration nor epidural analgesia proved superior to IV PCA on opioid consumption. Furthermore, epidural analgesia was noninferior to opioid group on pain scores. A possible explanation is that the epidural solution used in our institution contains a substantial amount of opioid (2 mcg/ml of fentanyl) which was included in the total opioid consumption. And as mentioned above, TAP infiltration does not prevent the visceral component of abdominal pain which probably prompted some opioid use by patients—thus compromising our ability to demonstrate superiority. And finally, either approach can produce uneven coverage or patchy infiltration which will prompt patients to use intravenous opioids and making it difficult to demonstrate the superiority.

Opioid-related side effects including ileus and postoperative nausea and vomiting were similar, presumably because opioid consumption was similar. NSAID and steroid drugs are usually scheduled, rather than provided per patient request, making it difficult to demonstrate a difference in a retrospective study.

Existing literature on epidural analgesia and hospital length-of-stay are inconsistent [21, 22]. Previous studies demonstrated that epidural analgesia either decreased length-of-stay by reducing pain scores and duration of ileus, or prolonged length-of-stay by causing urinary retention and hypotension [22]. We are unaware of previous reports comparing TAP infiltration with epidural analgesia on hospital length-of-stay. We found that Epidural patients stayed about two days longer in the hospital than either TAP or IV PCA patients. This is a highly clinically important prolongation and corresponds to an increase in hospital cost per stay of approximately $3,000 [23].

As with any all-retrospective studies, our ability to adjust for potential confounding is limited to available data. Although we accounted for potential confounding effects of eighteen patient and surgical factors, residual bias due to uncontrolled confounding variables remains possible. For example, anesthesiologists may have preferred epidural catheter placement in sicker patients, scheduled longer surgery, or having longer surgical incisions—thus selecting patients likely to stay longer in the hospital. We were unable to evaluate postoperative ambulation, but patients with epidural analgesia are likely to be less mobile than the other groups which may have prolonged the duration of hospitalization while possibly reducing pain scores and opioid consumption. In our database only 10 point numeric rating pain scores were recorded, have no detailed data for pain measurement i.e the American Pain Society Patient Outcomes Questionnaire-revision 1 and Pain Out have established validated tools for analyzing somatic and psychosocial components of pain. Potential residual bias limits our ability to make causal conclusions, which are most reliably derived from randomized trials. Consequently, the associations we report should not be considered evidence of a causal relationship.

## Conclusion

TAP infiltration and epidural analgesia did not differ in pain and opioid consumption and did not have worse pain scores than IV PCA after abdominal surgery. Our retrospective analysis suggests that TAP infiltration with liposomal bupivacaine is a reasonable alternative to epidural analgesia, and to IV PCA. A large randomized trial comparing these techniques is justified.
