# Correction: Comparison of Transversus Abdominis Plane Infiltration with Liposomal Bupivacaine versus Continuous Epidural Analgesia versus Intravenous Opioid Analgesia

**DOI:** 10.1371/journal.pone.0163687

**Published:** 2016-09-22

**Authors:** Sabry Ayad, Rovnat Babazade, Hesham Elsharkawy, Vinayak Nadar, Chetan Lokhande, Natalya Makarova, Rashi Khanna, Daniel I. Sessler, Alparslan Turan

The Competing Interests Statement is incorrect. The correct Competing Interests Statement is as follows:

Sabry Ayad is a paid consultant for Pacira Pharmaceuticals, Inc. There are no patents, products in development or marketed products to declare, and this does not alter the authors' adherence to PLOS ONE policies on sharing data and materials.
